# Foods, nutrients or whole diets: effects of targeting fish and LCn3PUFA consumption in a 12mo weight loss trial

**DOI:** 10.1186/1471-2458-13-1231

**Published:** 2013-12-26

**Authors:** Linda C Tapsell, Marijka J Batterham, Karen E Charlton, Elizabeth P Neale, Yasmine C Probst, Jane E O’Shea, Rebecca L Thorne, Qingsheng Zhang, Jimmy Chun Yu Louie

**Affiliations:** 1 Smart Foods Centre, University of Wollongong, Northfields Avenue, Wollongong NSW 2522, Australia

**Keywords:** Randomised controlled trial, LCn-3PUFA, Fish, Weight loss

## Abstract

**Background:**

There is some evidence in the literature that emphasising fish consumption may assist with weight loss. The aim was to assess the effects of advice to consume 2 fish meals per week in a weight loss diet.

**Methods:**

A parallel randomised placebo-controlled trial was conducted in 118 obese Australian adults (mean BMI ± SD 31.3 ± 3.5 kg/m^2^; mean age ± SD 45 ± 10 y; 28% male). Participants received low calorie dietary advice + placebo (1 g olive oil; Control), low calorie dietary advice emphasising fish + placebo (Fish), or low calorie dietary advice emphasising fish diet + LCn3PUFA supplements (Fish + S). Individualised advice targeted 2 MJ energy deficit (30%E fat, 45%E carbohydrate and 25%E protein) with or without two servings (180 g) fatty fish/wk.

**Results:**

All groups lost weight at 12 months (Control −4.5 kg vs. Fish −4.3 kg vs. Fish + S −3.3 kg; *p* < 0.001) and percentage body fat (Control: -1.5% vs. Fish: -1.4% vs. Fish + S: -0.7%; *p* < 0.001) but there were no significant differences between groups. Cardiovascular disease risk factors changed as expected from weight loss.

**Conclusions:**

Advice to consume 2 fish meals per week did not enhance the effects on weight loss of a healthy low calorie diet.

**Trial registration:**

ACTRN12608000425392.

## Background

Dietary guidance draws on evidence of health effects from foods, nutrients and whole diets [1,2], yet that these are all inter-related. There are calls for a greater emphasis on food in the construction of dietary guidelines [3] and this has implications for nutrition research, particularly with the concurrent need to address the obesity problem. Weight management reflects total energy intake which is delivered by the whole diet. Evidence for differential effects of single foods may be difficult to demonstrate when total dietary energy is kept below requirements. In addition, trial participants may choose foods and dietary patterns that are not fully compliant with the treatment, and in many cases the results reflect effects of dietary advice strategies, not of the foods, diets or nutrients targeted, (for example see [4]). Nevertheless, evidence based statements can be found drawing on literature that shows effects and associations between food intakes and health outcomes including weight management [5].

LCn3PUFA is an example of a key nutrient that has been implicated in weight loss. Observational studies show a relationship between LCn3PUFA status and a healthier BMI [6], and body composition [7]. Small, short term experimental studies show that a dose of 1.8 g LCn3PUFA /day can preferentially decrease adiposity [8] and increase fat oxidation [9]. This research suggests plausible mechanisms of nutrient action, but this research has not translated to consistent clinical outcomes. One study has shown that doses of around 3 g LCn3PUFA /day can enhance weight loss [10] but others testing the same dose [11] or as 1.4% energy [12] have not found no effect. From a food perspective, LCn3 PUFA are primarily found in fish, so further research examining effects of fish consumption may be informative. One short term study found that eating oily fish or taking supplements may be equally beneficial in achieving a greater weight loss, at least in fish eating populations [13].

Mechanistic and short term studies provide evidence at a proof of concept level [14], but for dietary guidance, weight loss maintenance is the major health goal. Long term dietary trials are difficult to conduct and a loss to follow up can be expected [15] with publications showing a range of reporting [16]. In addition, translating the concept from mechanistic and short term studies also means testing an appropriate amount of food and/or supplements which might align with current recommendations. In terms of LCn3PUFA and fish, this is about 600 mg LCn3PUFA or 2 fish meals/week [17]. The aim of the study reported here was to assess the effects on long term weight loss of advice to consume low calorie diets emphasising 2 fish meals per week with or without active supplements delivering equivalent amounts of LCn3PUFA.

## Methods

### Study design

A 12 month randomised controlled trial was conducted between 2009–2010 with overweight adults in three parallel diet advice arms: low calorie + placebo (control); low calorie + fish + placebo (Fish); and low calorie + fish + eicosapentaenoic acid (EPA)/docosahexanoic acid (DHA) supplements (Fish + S). The second intervention arm served as an enhancement strategy in case of poor compliance to fatty fish consumption over the year. The primary outcomes were change in body weight, BMI and percentage body fat. Secondary outcomes included change in cardiovascular disease risk factors. Recruitment was by media advertisements and emails sent by the research team throughout Wollongong, a major coastal city 70 km south of Sydney, Australia. This study was conducted according to Declaration of Helsinki guidelines and procedures were approved by the University of Wollongong Human Research Ethics Committee. Written informed consent was obtained from all participants. This trial is registered at http://www.anzctr.org.au (ID: ACTRN12608000425392).

### Randomisation and blinding

A researcher independent of the subject interface undertook the randomisation of participants into diet groups (stratified by sex and block randomised; nQuery Advisor V 7.0, Statistical solutions, Cork, Ireland) and the code was kept from the researchers collecting dietary data and delivering treatment. Supplements were coded off-site. Different dietitians collected dietary data and provided dietary advice. Those providing advice were necessarily aware of diet category but not of supplement allocation.

### Participants

The study sample comprised middle aged (mean ± SD age 45 ± 10 y) obese adults (mean ± SD BMI 31.3 ± 3.5 kg/m^2^). Inclusion criteria included: age between 18 to 60 years, BMI between 25 to 37 kg/m^2^, waist circumference > 94 cm (men) or > 80 cm (women), otherwise healthy. Individuals with major illnesses, diabetes mellitus, LDL ≥ 6 mmol/L, food allergies or habits inhibiting compliance, low literacy, inadequate conversational English, and those who are already taking fish oil supplements, unable to undertake study requirements, pregnant/lactating, not weight stable (within 3 kg) in the past 6 months or on a weight-loss diet were excluded from participation. Participants were not paid.

### Dietary intervention

The intervention addressed dietary intake at the, food (fish), key nutrient (LCn3PUFA) and whole diet (low calorie) level. The fatty fish target and LCn3PUFA equivalent reference value were based on materials in the Nutrient Reference Values (NRVs) report for Australia and New Zealand [17]. These suggested two servings (180 g) of fatty fish per week and a dietary intake for LCn3 PUFA based on the 90^th^ percentile of population consumption levels (i.e. 610 mg/day for men and 430 mg/day for women) [17]. All groups were advised on a hypocaloric diet (2 MJ energy below estimated energy requirements estimated by the Mifflin equation [18] with 1.25 physical activity factor), targeting 25% energy from protein, 45% energy from carbohydrate and 30% energy from fat. Diets referred to low fat staple foods (fruit, vegetables, cereals, lean meat, low fat milk and yoghurt) and small amounts of nuts, seeds, spreads and oils. Food groups were congruent with the Australian Guide to Healthy Eating [19], and formed the basis of the standardised procedure for both diet groups and the template of the diets sheets. Participants in the Fish and Fish + S advice groups were specifically encouraged to consume 180 g fatty fish per week. Participants in the control group were not given specific advice regarding fish intake, but were instructed to consume prescribed amounts of foods rich in protein, of which fish was an option. Dietary education was supported by print resources outlining methods of cooking the appropriate fish and recipes. The Control and Fish groups were given placebo capsules (1 g olive oil per day) and the Fish + S group the active supplements (420 mg EPA + 210 mg DHA; Blackmore’s Promega Heart, Blackmores Australia). Dietary education (1 hour) and follow-up (30 min) with one of seven experienced dietitians was provided at 0, 1, 2, 3, 6, 9, and 12 months with written materials and monthly newsletters to support compliance. Accredited Practising Dietitians collected dietary data at 0, 3 and 12 months via a validated diet history interview (DH) [20]. Dietary data was analysed using the FoodWorks software system (Version 6, 2009, Xyris Software, Spring Hill, QLD, Australia). Reported food intake data was converted to intakes of energy and macronutrients using the AUSNUT1999 [21] and AusFood2001 databases (revision 11, from FoodWorks 2009 version 6, Xyris Software, Spring Hill, QLD, Australia). Reported LCn3PUFA intakes were assessed separately using the AUSNUT2007 database [22]. The percentage fish in each dish or product was calculated based on a validated approximation of canned fish labels [23] and standardized recipe data. Habitual physical activity at baseline and 12 months was assessed by questionnaire [24]. Advice was given to all groups to walk for 30 minutes three days per week.

### Measurement of anthropometric and biochemical variables

Height was measured using a stadiometer without shoes. Body weight was measured in an upright position in minimal clothing, without shoes using digital scales with a bioelectrical impedance component (Tanita TBF-622; W.W. Wedderburn Pty Ltd, Ingleburn, NSW, Australia) at baseline and every 3 months until 12 months. Percentage body fat was measured using DEXA (Hologic QDR 4500; Hologic Inc. Bedford MA) from most participants at baseline (*n* = 104), 3 months (*n* = 66) and 12 months (*n* = 46).

Fasting blood samples taken at 0, 3, 6, 9 and 12 months were sent to a quality assured laboratory (Southern IML Pathology) to measure total cholesterol, HDL, LDL, triglycerides, insulin, and glucose. Leptin was assessed by the same laboratory for samples collected at 0, 3 and 12 months. Erythrocyte fatty acid concentrations at 0, 3 and 12 months, a measure of dietary compliance [25], were analysed at Australian Research Laboratories (now Healthscope Pathology), Melbourne, Australia. The omega-3 index was calculated as erythrocyte [EPA + DHA]/[total fatty acids] × 100% [26]. Insulin resistance was assessed by HOMA equations [27]. Blood pressure was measured using a medical grade blood pressure monitor (Dinamap XL Vital Signs Monitor). Participants sat for 10 mins then three readings were taken from the same arm, and the mean of the three readings was used as the final reading.

### Compliance to study protocol

Compliance to fish and supplement recommendations was calculated for all participants who remained in the trial at 12 months. In order to calculate compliance to fish recommendations, an average of each participant’s fish intake from all available diet histories was taken, as described by Alhassan *et al*[28]. Compliers were defined as consuming < 180 g fish/week for the control group, consuming ≥ 180 g fish/week for the Fish group, and either consuming ≥ 180 g fish/week or taking ≥ 90% of the supplements in the Fish + S group.

### Power calculations

**Figure 1:**
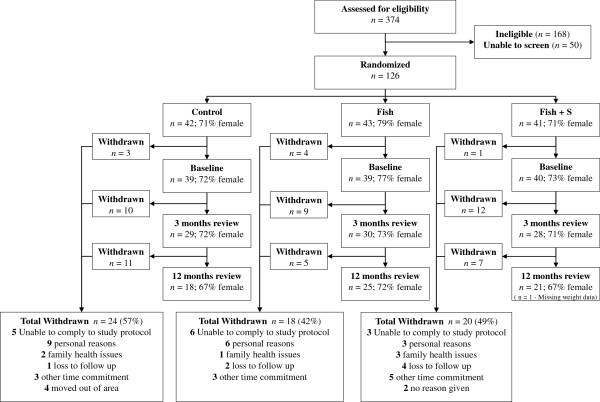
The flow of study (CONSORT diagram).

Using data from a previous study [29] and an expected weight change of −2.6 kg in the control and −4.7 kg in the intervention groups, we required 27 participants in each group to achieve statistical significance (α = 0.05, β = 0.9). As shown in Figure 1, we enrolled 126 people, from an initial pool of 374 volunteers. Eight participants withdrew from the study before treatment so their data were excluded from all analyses.

### Statistical analysis

All statistical analyses were performed using IBM SPSS (version 17.0 and 19.0, IBM Australia, Lane Cove, NSW, Australia). Analysis of the primary outcome (i.e. weight) was performed on an *intention to treat* basis with the 118 participants who provided baseline data, using a linear mixed model. Several approaches were taken to investigate the effect of missing data on the primary outcome. These included multiple imputation (using PROC MI in SAS V9.2, SAS Inc. Cary, NC), last observation carried forward (LOCF), and baseline observation carried forward (BOCF). These methods are the most commonly used in weight loss studies [30]. Completers only (*n* = 63) analyses were also performed.

Secondary outcomes were analysed using linear mixed model, using data on all participants. Covariates were included in the models where biologically appropriate, as noted in the results section. Intra-class correlations and chi square analyses were used to examine any potential therapist effect, and none was noted. Statistical significance was set at *p* < 0.05.

## Results

**Figure 2:**
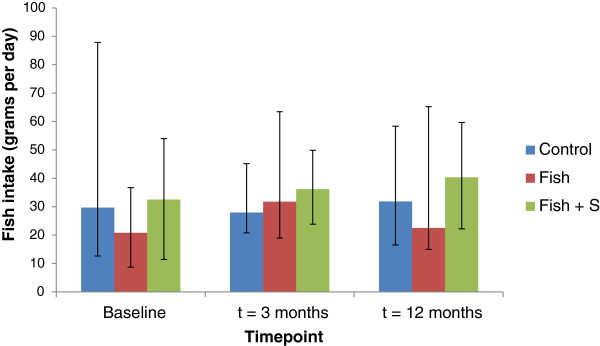
Fish consumption at baseline, 3 months and 12 months.

After the 3 month intensive phase n = 87 participants remained in the study and at the 12 month follow up data was available for n = 64 (weight data missing for n = 1). Reasons for attrition are shown in Figure 1. Of the 12 mo completers, 57% were judged to be compliant, 39% (n = 7) for the control group who reported <180 g fish/week, 48% (n = 12) for the Fish group who reported ≥180 g fish/week, and 85% (n = 17) for the Fish + S group who reported ≥180 g fish/week or ≥90% supplements. Actual fish consumption was highly variable (Figure 2). There was no difference between groups in dropout rates (log ‘rank test, *p* = 0.872), and the characteristics of participants who withdrew were similar to the completers (Table 1). There were no adverse events, and no changes in medications. Prior to randomization, participants reported plausible usual dietary intakes for the study population, including a median intake of 370 mg (men) and 290 mg (women) LCn3PUFA per day, more than half that of the suggested dietary targets (610 mg/day for men and 430 mg/day for women). Physical activity scores increased over 12 month, with no significant difference between groups (One-way ANOVA, *p* = 0.673).

**Table 1:** Subject characteristics at baseline

**Variable**	**All subjects who has baseline data**	**Completers**	**Dropouts**	***p *** **value**^**#**^	**Compliers**	**Non-compliers**	***p *** value*
*n*	118	63	55	-	36	27	-
Age (years)	45.1 ± 8.4	45.4 ± 9.2	44.9 ± 7.4	0.751	47.1 ± 8.3	43.1 ± 10.0	0.092
Male (%)	26.3	31.7	20.0	0.148	30.6	33.3	0.815
Weight (kg)	88.6 ± 12.1	88.5 ± 12.5	88.7 ± 11.6	0.921	89.5 ± 12.2	87.1 ± 13.1	0.444
BMI (kg/m^2^)	31.2 ± 3.5	30.9 ± 3.6	31.6 ± 3.4	0.302	31.6 ± 3.5	29.9 ± 3.5	0.069
Smokers (%)	5.9	6.3	5.5	0.837	8.3	3.7	0.456
Education level TAFE or above (%)	64.4	68.3	60.0	0.350	55.6	85.2	0.012
Labour intensive job (%)^1^	35.0	33.3	37.0	0.713	36.1	29.6	0.359
Taking regular medication (%)	26.3	20.6	32.7	0.137	22.2	18.5	0.719
Country of birth (%)							
*Oceania*	83.6	87.1	79.6		85.7	88.9	
*Europe*	12.1	9.7	14.8	0.552	11.4	7.4	0.858
*Others*	4.3	3.2	5.6		2.9	3.7	

Values are mean ± SD or percentages. *p* values obtained using ANOVA for continuous variables and Pearson’s Chi’s square for categorical variables.

^#^*p* value for differences between completers and dropouts.

^*^p value for differences between compliers and non-compliers.

^1^Labour intensive jobs included: carer, home duties, retail and trade professionals.

### Effects on weight loss and body fat

**Figure 3:**
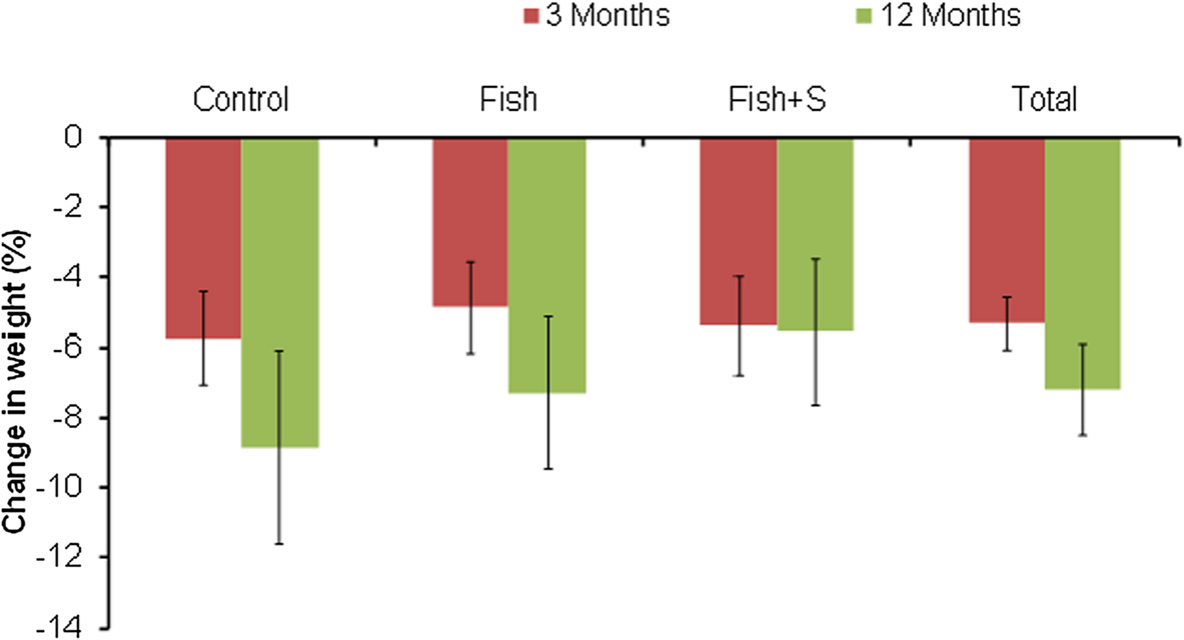
Change in % body weight by group at 3 and 12 months.

In the intention to treat analysis (Table 2) based on the linear mixed model, all groups lost weight at 12 months (Control −4.5 kg vs. Fish −4.3 kg vs. Fish + S −3.3 kg; *p* < 0.001) and percentage body fat significantly decreased (Control: -1.5% vs. Fish: -1.4% vs. Fish + S: -0.7%; *p* < 0.001). There were no significant differences between groups for these variables and this result remained after adjustment for EPA + DHA status (Figure 3). All other methods of analysis for the primary outcome similarly showed a significant time effect, with no significant group differences, and restricting the analysis to completers only did not significantly change the results. After stratifying the analysis based on compliance, there was no significant weight loss among non-compliers regardless of dietary assignment (time effect = 0.653; group effect = 0.371; interaction = 0.972). Significant weight loss was observed among compliers (87.7 kg at baseline vs. 79.7 kg at 12 months; time effect = 0.034); but there was no difference between groups (group effect = 0.299; interaction = 0.996). The mean weight loss at three months was greater in those who completed the 12 months than those who did not (−5.2 vs. -3.2 P = 0.005).

**Table 2:** Weight of participants at baseline, 3 months and 12 months

	**Control**			**Fish**			**Fish + S**			***p *** **values**		
	**Baseline**	**3 months**	**12 months**	**Baseline**	**3 months**	**12 months**	**Baseline**	**3 months**	**12 months**	**Time**	**Group**	**Interaction**
***Mixed model with all available data only***												
*n*	39	29	18	39	30	25	40	28	20	-	-	-
Mean ± SD	88.5 ± 12.7	84.2 ± 12.6	81.7 ± 14.1	90.0 ± 12.2	84.8 ± 12.3	80.9 ± 12.7	88.5 ± 11.4	82.9 ± 11.7	84.3 ± 11.7	< 0.001	0.682	0.326
										< 0.001^‡^	0.617^‡^	0.338^‡^
***Mixed model with missing values imputed***												
*n*	39	39	39	39	39	39	40	40	40	-	-	-
Mean ± SD	88.5 ± 12.7	83.7 ± 12.0	82.9 ± 13.0	90.0 ± 12.2	86.0 ± 12.3	84.7 ± 13.2	88.5 ± 11.4	83.8 ± 11.3	83.7 ± 11.3	< 0.001	0.767	0.803
***LOCF***^***#***^												
*n*	39	39	39	39	39	39	40	40	40	-	-	-
Mean ± SD	88.5 ± 12.7	84.6 ± 12.1	83.9 ± 12.7	90.0 ± 12.2	86.7 ± 12.8	85.7 ± 13.6	88.5 ± 11.4	85.2 ± 11.5	85.3 ± 11.7	< 0.001	0.805	0.457
***BOCF***^***#***^												
*n*	39	39	39	39	39	39	40	40	40	-	-	-
Mean ± SD	88.5 ± 12.7	84.6 ± 12.1	84.8 ± 13.1	90.0 ± 12.2	86.7 ± 12.8	86.0 ± 14.0	88.5 ± 11.4	85.2 ± 11.5	86.0 ± 11.2	< 0.001	0.840	0.348
***Completers only***^***#***^												
*n*	18	18	18	25	25	25	20	20	20	-	-	-
Mean ± SD	89.5 ± 13.9	83.2 ± 13.0	81.7 ± 14.1	87.1 ± 11.8	82.5 ± 11.8	80.9 ± 12.7	89.4 ± 12.3	84.2 ± 11.4	84.3 ± 11.7	< 0.001	0.799	0.141

^‡^Adjusted for omega-3 index (red blood cell EPA + DHA).

^***#***^*p* value calculated by repeated measures analysis of variance based on data from five time points (0, 3, 6, 9 and 12 months).

LOCF last observation carried forward; BOCF baseline observation carried forward.

### Effects on cardiovascular disease risk factors and biomarkers

All groups showed improvements in fasting insulin, HOMA-IR and triglycerides (Table 3). Systolic blood pressure, fasting glucose, total cholesterol and leptin levels decreased for the entire study sample, with no difference between groups. There was a significant time by group interaction for self-reported intake of LCn3PUFA (Table 4, *p* = 0.034), confirmed by a significant group difference in erythrocyte omega-3 index (Table 3, *p* = 0.009). This effect was primarily due to changes in the Fish + S group, and *post hoc* analysis revealed a significant difference between the Fish and Fish + S groups (Fish: 3.4 ± 1.0% vs. Fish + S: 4.4 ± 1.6%; *p* = 0.007).

**Table 3:** Body mass index, % body fat and clinical assessments at baseline, 3 months and 12 months

**Variable**	**Control**		**Fish**		**Fish + S**		**Difference between groups**	
	***n***	**Value**	***n***	**Value**	***n***	**Value**	**Effect**	***p *** value*
***Body mass index (kg/m***^***2***^ ***)*** **†**								
Baseline	39	30.9 ± 3.5	39	31.4 ± 3.6	40	31.6 ± 3.4	*Time*	< 0.001
3 months	29	29.3 ± 3.4	30	29.5 ± 3.6	28	29.6 ± 3.1	*Group*	0.376
12 months	18	28.0 ± 3.6	25	28.2 ± 3.4	20	29.8 ± 3.5	*Interaction*	0.370
***Body fat (%)*** **†#**								
Baseline	34	36.7 ± 7.3	36	38.0 ± 7.5	36	36.0 ± 8.7	*Time*	< 0.001
3 months	21	33.4 ± 7.7	25	35.5 ± 7.6	20	33.0 ± 10.3	*Group*	0.595
12 months	14	34.4 ± 7.6	16	37.4 ± 8.1	16	34.0 ± 10.2	*Interaction*	0.369
***Fasting glucose level (mmol/L)*** **†**								
Baseline	37	5.0 ± 0.5	39	5.1 ± 0.5	39	5.0 ± 0.6	*Time*	0.009
3 months	27	4.9 ± 0.5	30	5.0 ± 0.6	28	4.7 ± 0.6	*Group*	0.585
12 months	17	5.1 ± 0.4	24	4.9 ± 0.7	21	4.8 ± 0.4	*Interaction*	0.350
***Fasting insulin level (mU/L)*** **†**‡								
Baseline	37	9.1 (7.7 – 12.9)	39	9.6 (7.4 – 13.8)	39	12.0 (8.5 – 17.0)	*Time*	0.001
3 months	27	8.7 (7.1 – 11.5)	29	9.1 (7.4 – 12.2)	28	10.4 (7.2 – 13.0)	*Group*	0.360
12 months	18	8.5 (6.3 – 10.5)	24	8.2 (6.2 – 11.1)	21	9.2 (6.5 – 13.1)	*Interaction*	0.086
***Fasting total cholesterol (mmol/L)*** **†**								
Baseline	37	5.3 ± 1.2	39	5.3 ± 0.8	39	5.2 ± 0.9	*Time*	< 0.001
3 months	27	4.8 ± 0.8	30	5.0 ± 0.8	28	5.1 ± 0.9	*Group*	0.309
12 months	18	4.6 ± 0.7	24	5.2 ± 1.0	21	5.4 ± 0.9	*Interaction*	0.098
***Fasting triglycerides (mmol/L)*** **†**‡								
Baseline	37	1.4 (0.9 – 2.0)	39	1.3 (1.0 – 1.8)	39	1.3 (1.0 – 2.1)	*Time*	< 0.001
3 months	27	1.3 (0.9 – 1.6)	30	1.3 (1.0 – 1.5)	28	1.1 (0.7 – 1.7)	*Group*	0.661
12 months	18	1.0 (0.7 – 1.5)	24	1.1 (0.9 – 1.4)	21	1.0 (0.6 – 1.5)	*Interaction*	0.122
***Fasting HDL cholesterol (mmol/L)*** **†**								
Baseline	37	1.5 ± 0.4	39	1.5 ± 0.4	39	1.4 ± 0.4	*Time*	< 0.001
3 months	27	1.5 ± 0.3	30	1.4 ± 0.3	28	1.4 ± 0.5	*Group*	0.566
12 months	18	1.4 ± 0.3	24	1.4 ± 0.4	21	1.5 ± 0.4	*Interaction*	0.739
***Fasting LDL cholesterol (mmol/L)*** **†**								
Baseline	37	3.1 ± 1.1	39	3.1 ± 0.8	38	3.1 ± 0.8	*Time*	0.001
3 months	27	2.7 ± 0.8	30	3.0 ± 0.8	28	3.1 ± 0.7	*Group*	0.242
12 months	18	2.7 ± 0.8	24	3.2 ± 0.9	21	3.4 ± 0.9	*Interaction*	0.091
***Fasting leptin (mg/mL)*** ¶‡								
Baseline	37	21.5 (11.7 – 31.3)	38	19.9 (12.3 – 29.6)	39	18.0 (10.4 – 30.9)	*Time*	< 0.001
3 months	27	11.8 (7.2 – 20.5)	30	14.7 (7.2 – 20.8)	28	14.1 (7.0 – 22.6)	*Group*	0.682
12 months	18	10.7 (5.6 – 16.3)	24	14.1 (7.2 – 23.3)	21	16.7 (6.5 – 27.3)	*Interaction*	0.083
***Systolic blood pressure (mmHg)***								
Baseline	37	126 ± 16	39	123 ± 16	38	124 ± 16	*Time*	0.002
3 months	27	125 ± 12	30	120 ± 16	26	116 ± 12	*Group*	0.673
12 months	16	120 ± 11	23	128 ± 17	20	120 ± 13	*Interaction*	0.118
***Diastolic blood pressure (mmHg)***								
Baseline	37	74 ± 10	39	72 ± 9	38	72 ± 9	*Time*	0.079
3 months	27	74 ± 9	30	72 ± 9	26	70 ± 8	*Group*	0.990
12 months	16	72 ± 7	23	77 ± 10	20	74 ± 9	*Interaction*	0.112
***HOMA-IR***								
Baseline	37	1.6 ± 1.1	39	1.5 ± 0.7	39	1.7 ± 0.7	*Time*	< 0.001
3 months	27	1.3 ± 0.7	29	1.5 ± 0.9	28	1.4 ± 0.5	*Group*	0.326
12 months	17	1.2 ± 0.5	24	1.2 ± 0.5	21	1.3 ± 0.5	*Interaction*	0.300
Omega-3 index (%)^**^								
Baseline	36	4.0 ± 1.2	38	3.5 ± 1.1	39	3.6 ± 1.1	*Time*	0.240
3 months	26	3.6 ± 2.0	30	3.6 ± 1.2	28	4.5 ± 1.7	*Group*	0.009
12 months	15	3.7 ± 0.9	23	3.4 ± 1.0	21	4.4 ± 1.6	*Interaction*	0.116

Values are expressed as mean ± SD or median (interquartile range).

HOMA-IR, homeostasis model assessment of insulin resistance.

*Linear mixed model, significance at *p* = 0.05.

¶Leptin adjusted for body fat.

†*p* value based on five time points (0, 3, 6, 9 and 12 months).

#Body fat assessed by DEXA.

‡Ln transformed.

^**^Erythrocyte[EPA + DHA]/[total fatty acids] × 100%.

**Table 4:** Reported daily energy, macronutrient and fibre intakes at baseline, 3 months and 12 months

**Variable**	**Control**		**Fish**		**Fish + S**		**Difference between groups**	
	***n***	**Value**	***n***	**Value**	***n***	**Value**	**Effect**	***p *** value*
***Energy (kJ)***								
Baseline	39	9230 ± 2930	39	10190 ± 3970	40	10480 ± 3320	*Time*	< 0.001
3 months	29	6600 ± 1510	29	6640 ± 1550	28	6350 ± 1240	*Group*	0.581
12 months	18	7330 ± 1750	25	6760 ± 1750	21	6700 ± 1490	*Interaction*	0.185
***%E from protein***								
Baseline	39	19.5 ± 3.5	39	17.8 ± 3.2	40	18.1 ± 3.1	*Time*	< 0.001
3 months	29	23.1 ± 4.1	29	22.4 ± 3.2	28	21.9 ± 2.7	*Group*	0.075
12 months	18	22.5 ± 3.9	25	21.2 ± 3.8	21	21.6 ± 3.2	*Interaction*	0.833
***%E from fat***								
Baseline	39	32.4 ± 5.3	39	35.5 ± 5.9	40	34.9 ± 6.5	*Time*	< 0.001
3 months	29	25.0 ± 6.2	29	27.0 ± 6.3	28	24.9 ± 4.3	*Group*	0.015
12 months	18	24.4 ± 5.6	25	29.1 ± 7.1	21	26.9 ± 5.8	*Interaction*	0.366
***%E from saturated fat***								
Baseline	39	12.2 ± 3.0	39	12.7 ± 3.0	40	13.0 ± 4.0	*Time*	< 0.001
3 months	29	8.1 ± 2.2	29	8.0 ± 2.2	28	7.6 ± 1.6	*Group*	0.800
12 months	18	8.5 ± 2.9	25	8.7 ± 2.7	21	8.6 ± 2.7	*Interaction*	0.727
***%E from polyunsaturated fats***								
Baseline	39	5.2 ± 1.9	39	13.9 ± 3.1	40	13.6 ± 3.5	*Time*	0.570
3 months	29	4.4 ± 2.1	29	9.6 ± 2.5	28	9.3 ± 2.2	*Group*	< 0.001
12 months	18	3.9 ± 1.1	25	11.3 ± 3.8	21	9.9 ± 3.1	*Interaction*	0.153
***%E from monounsaturated fats***								
Baseline	39	12.0 ± 2.5	39	13.9 ± 3.1	40	13.6 ± 3.5	*Time*	< 0.001
3 months	29	9.8 ± 3.3	29	9.6 ± 2.5	28	9.3 ± 2.2	*Group*	0.075
12 months	18	9.3 ± 2.5	25	11.3 ± 3.8	21	9.9 ± 3.0	*Interaction*	0.039
***P:S ratio***								
Baseline	39	0.47 ± 0.24	39	0.50 ± 0.22	40	0.45 ± 0.19	*Time*	< 0.001
3 months	29	0.58 ± 0.31	29	0.88 ± 0.45	28	0.72 ± 0.29	*Group*	0.007
12 months	18	0.50 ± 0.21	25	0.78 ± 0.59	21	0.73 ± 0.42	*Interaction*	0.061
***Long chain n-3 polyunsaturated fatty acids (mg)*** †‡								
Baseline	39	292 (195 – 760)	39	310 (198 – 446)	40	310 (167 – 474)	*Time*	0.085
3 months	29	314 (178 – 456)	29	430 (219 – 917)	28	512 (272 – 706)	*Group*	0.595
12 months	18	393 (195 – 521)	25	421 (129 – 675)	21	355 (178 – 815)	*Interaction*	0.034
***%E from carbohydrates***								
Baseline	39	41.5 ± 6.0	39	40.8 ± 6.1	40	43.4 ± 6.8	*Time*	< 0.001
3 months	29	43.2 ± 10.9	29	44.7 ± 7.3	28	47.8 ± 5.5	*Group*	0.026
12 months	18	45.6 ± 6.0	25	43.4 ± 6.6	21	46.1 ± 4.9	*Interaction*	0.457
***%E from alcohol***								
Baseline	39	4.9 ± 5.2	39	4.3 ± 4.3	40	2.3 ± 3.4	*Time*	0.611
3 months	29	4.8 ± 4.8	29	3.5 ± 3.8	28	2.9 ± 4.3	*Group*	0.159
12 months	18	5.4 ± 6.0	25	4.0 ± 5.0	21	3.0 ± 3.9	*Interaction*	0.218
***Dietary cholesterol (mg)***								
Baseline	39	308 ± 135	39	322 ± 222	40	312 ± 113	*Time*	< 0.001
3 months	29	213 ± 68	29	211 ± 64	28	191 ± 64	*Group*	0.827
12 months	18	242 ± 89	25	191 ± 63	21	209 ± 73	*Interaction*	0.885
***Dietary fibre (g)***								
Baseline	39	25.7 ± 8.4	39	28.5 ± 9.4	40	28.6 ± 12.4	*Time*	0.008
3 months	29	23.9 ± 6.1	29	25.6 ± 5.9	28	26.1 ± 4.9	*Group*	0.535
12 months	18	25.2 ± 5.8	25	25.1 ± 6.5	21	25.3 ± 5.7	*Interaction*	0.855

Values are expressed as mean ± SD or median (interquartile range).

P:S ratio, polyunsaturated fatty acid to saturated fatty acid ratio.

*Linear mixed model, significance at *p* = 0.05.

†Ln transformed.

‡LC n-3 includes: 20:3n3 (eicosatrienoic acid), 20:5n3 (eicosapentaenoic acid), 22:5n3 (docosapentaenoic acid) and 22:6n3 (docosahexaenoic acid). Dietary data did not include supplementation.

## Discussion

Under free living conditions, advice emphasising 2 fatty fish meals a week did not enhance weight loss achieved through low calorie dietary advice alone. The addition of equivalent amounts of LCn3PUFA supplements slightly increased LCn3PUFA status but did not influence weight loss. The required energy deficit appeared met by the total diet in all treatment groups, making it difficult to attribute effects to advice on fish consumption, even if supplemented with their key nutrients (LCn3PUFA).

From a nutrient perspective, our results are consistent with other shorter term trials [11,12], but possibly for different reasons. The target LCn3PUFA intake was the Nutrient Reference Value for Australia and New Zealand of about 600 mg [31]. The higher biomarker status in the supplemented group suggested that supplements may assure better LCn3PUFA intakes than advice to consume fish, but this did not enhance weight loss. It may be that higher doses are required, although weight loss still was still achieved regardless of supplementation.

Factors that may have affected results, such as accounting for the n-6:n-3 ratio [32] did not change the outcomes. We used a similar approach to supplementation as other studies testing effects of dietary LCn3PUFA in ranges consumed in most populations (< 2 g/day) and using an olive oil placebo [33]. Our results were consistent with one of our previous trials [29], and while a similar study showed greater improvements in disease risk factors than ours [15], our study was longer (12 months vs. 24 weeks) and our sample was not as overweight, appeared to have lower levels of risk, and our target intake of LCn3PUFA was lower (0.42 g EPA + 0.21 g DHA vs. 1.3 g EPA + 2.9 g DHA). We acknowledge that low concentrations of erythrocyte EPA and DHA have been linked with a number of negative health effects [34] but this is a separate issue to weight loss in a low calorie dietary content.

The dietary advice was based on nutrient-rich core foods: vegetables, wholegrain cereals, fruits, lean meats and low fat dairy foods, and we controlled for advice on the total diet. Nevertheless, our results remained congruent with a similar study applying a more liberal *ad libitum* dietary advice strategy [12]. The considerable variation in fish intakes, demonstrated the difficulty in sustaining levels of fish consumption. The magnitude of effect of the intervention was good, similar to that of a 12 months multicentre trial comparing different dietary advice strategies [4]. Both studies showed no difference in weight loss between diet advice groups with a lack of compliance to actual dietary prescriptions. These studies confirm that dietary energy intake is the main factor in weight loss, but at the same time, the intention to treat analysis provides evidence for the effects of the dietary advice strategy.

As demonstrated in previous research [4], weight loss in our study was associated with significant improvements in a wide range of cardiovascular disease risk factors. In the sample as a whole, we observed significant reductions in systolic blood pressure, glucose, insulin, and insulin resistance. Triglycerides were not significantly reduced, but this was consistent with other findings [15]. The drop in leptin levels across the sample was congruent with reduced energy intakes, as would be expected [35] and did not differ by treatment.

There were limitations to the study. As a feature of long term ‘free living’ weight loss trials [15], the attrition rate is disappointing, although the level was within the range of reporting [16]. We applied state of the art statistical analyses and while we acknowledge some limitations [36] to the BOCF and LOCF methods, these are the most commonly used in the weight loss RCT literature [15]. The assumption that those who lost more weight are more likely to complete is also considered in the analysis. We saw little influence of attrition on results, drawing conclusions from the intention to treat analysis vs. completers only analysis, and between the characteristics of completers vs. dropouts.

The hypothesised effect was a difference in favour of the treatment group (fish emphasis) of a weight loss of 2.1 kg greater than the control (no fish emphasis). The results show that the confidence intervals for the between group differences in the completers do not include this value. In addition, the difference between the Control and Fish arm (−1.6 kg, 95% CI −5.1,1.8 in favour of control) in the completers did not support the alternate hypothesis that emphasising fish in the dietary advice has a greater weight loss effect. This was still the case with the Control and Fish + Supps (−2.7 kg 95% CI-6.4,0.9 kg) where we had enhanced the advice with fish oil supplements. The upper CI of both intervention arms shows a value that is not statistically significant. (Additional file 1: supplementary Tables S1 and S2 show similar lack of differences between completers for change in dietary intake and other outcomes at 12 months). A greater weight loss of 0.9 or 1.8 kg in the treatment groups compared with the control may be too small to be considered clinically relevant after 12 mo. Future studies could use our results to estimate sample sizes for equivalence or use the variance estimates to determine difference needed for effect.

A major strength of our study was that the trial extended to 12 months follow up [37], with regular monitoring and continual engagement with the participants to motivate sustained dietary change. Compliance with the supplement regimen was confirmed with erythrocyte fatty acid concentrations. By basing dietary advice in all groups on core foods, we addressed the lack of dietary controls and variation in food sources which often hampers studies of dietary fat [38].

## Conclusions

Advice to consume 2 fish meals per week did not appear to enhance the effects on weight loss of a healthy low calorie diet, however further studies with greater power may be required to make this conclusion. In considering the relative roles of nutrients, food and whole diets [3], we conclude that the whole diet may the most tangible nutritional parameter in deriving evidence for food effects in weight loss studies.

## Competing interests

The authors declare they have no competing interests.

## Authors’ contributions

LT - designed the research, wrote the paper and had primary responsibility for the final manuscript. MB – contributed to study design, performed the randomisation process and undertook statistical analysis. KC - contributed to study design and contributed to manuscript. YP - provided essential materials for the construction of databases, contributed to manuscript and conducted research at the clinical interface. EN - provided essential materials for the construction of databases, contributed to manuscript and conducted research at the clinical interface. JO - conducted the research at the clinical interface, contributed to manuscript and provided key summaries and preparation of data. RT - conducted research at the clinical interface, contributed to manuscript and assisted in the preparation of data and submission of the final document. QS - Conducted research at the clinical interface. JL – contributed to final manuscript and undertook statistical analysis. All authors read and approved the final manuscript.

## Pre-publication history

The pre-publication history for this paper can be accessed here:

http://www.biomedcentral.com/1471-2458/13/1231/prepub

## Supplementary Material

Additional file 1: Table S1 12-month change from baseline (95% Confidence Intervals) in dietary variables by group, amongst completers only. **Table S2.** 12-month change from baseline (95% Confidence Intervals) in outcome variables by group amongst completers only. Click here for file
