# Supplementary material for: Foods, nutrients or whole diets: effects of targeting fish and LCn3PUFA consumption in a 12mo weight loss trial
Source: BMC Public Health. 2013 Dec 26;13:1231. doi: 10.1186/1471-2458-13-1231 (PMC3890608; doi:10.1186/1471-2458-13-1231)
Supplement: Additional file 1: Table S1 — 12-month change from baseline (95% Confidence Intervals) in dietary variables by group, amongst completers only. Table S2. 12-month change from baseline (95% Confidence Intervals) in outcome variables by group amongst completers only. [file 1471-2458-13-1231-S1.docx]

**Additional file 1:**

**Supplemental Table S1: 12-month change from baseline (95% Confidence Intervals) in dietary variables by group, amongst completers only**

| ***Variable*** | ***Control*** | ***Fish*** | ***Fish + S*** | ***P value (ANOVA)*** |
| --- | --- | --- | --- | --- |
| N | 18 | 25 | 21 | - |
| Δenergy (kJ) | -2955 (-4388, -1521) | -2505 (-3332, -1679) | -3956 (-5419, -2493) | 0.193 |
| Δ%E from protein | 3.0 (1.2, 4.8) | 3.7 (2.1, 5.2) | 3.3 (1.9, 4.7) | 0.818 |
| Δ%E from fat | -8.1 (-11.4, -4.9) | -5.5 (-8.5, -2.6) | -8.9 (-12.6, -5.1) | 0.270 |
| Δ%E from saturated fat | -4.7 (-6.2, -3.2) | -3.6 (-4.5, -2.7) | -4.1 (-6.1, -2.1) | 0.572 |
| Δ%E from polyunsaturated fat | -0.6 (-1.5, 0.2) | 0.4 (-0.9, 1.8) | -0.7 (-2.6, 1.2) | 0.406 |
| Δ%E from monounsaturated fat | -2.6 (-4.3, -1.0) | -2.3 (-4.1, -0.5) | -4.6 (-6.5, -2.6) | 0.149 |
| ΔP:S ratio | 0.13 (0.02, 0.25) | 0.28 (0.05, 0.51) | 0.24 (0.09, 0.40) | 0.505 |
| ΔLCn3PUFA (mg) | -244.6 (-528.4, 39.1) | 27.9 (-138.2, 194.0) | 56.8 (-122.6, 236.2) | 0.083 |
| Δ%E from carbohydrate (%) | 5.0 (1.5, 8.5) | 1.8 (-1.4, 5.0) | 4.4 (0.7, 8.2) | 0.338 |
| Δ%E from alcohol (%) | -0.5 (-2.2, 1.1) | -0.6 (-1.7, 0.6) | 0.2 (-0.7, 1.2) | 0.560 |
| Δdietary cholesterol (mg) | -110 (-187, -34) | -92 (-133, -51) | -103 (-158, -49) | 0.885 |
| Δdietary fibre (g) | -1.0 (-4.9, 2.8) | -3.0 (-6.6, 0.6) | -4.8 (-11.0, 1.5) | 0.537 |

**Supplemental Table S2: 12-month change from baseline (95% Confidence Intervals) in outcome variables by group amongst completers only**

| ***Variable*** | ***n*** | ***Control*** | ***n*** | ***Fish*** | ***n*** | ***Fish + S*** | ***P value (ANOVA)*** |
| --- | --- | --- | --- | --- | --- | --- | --- |
| BMI (kg/m^2^) | 18 | -2.7 (-3.6, -1.8) | 25 | -2.2 (-2.9, -1.6) | 20 | -1.8 (-2.5, -1.1) | 0.244 |
| % Body Fat | 14 | -2.3 (-4.2, -0.3) | 16 | -2.6 (-4.0, -1.2) | 16 | -1.2 (-2.4, -0.0) | 0.335 |
| Fasting glucose  (mmol/L) | 17 | 0.2 ( -0.2, 0.5) | 24 | -0.2 (-0.5, 0.0) | 21 | -0.0 (-0.3, 0.2) | 0.115 |
| Fasting insulin (pmol/L) | 18 | -1.8 (-3.6, 0.1) | 24 | -1.5 (-3.0, -0.1) | 21 | -2.0 (-3.6, -0.5) | 0.895 |
| Fasting total cholesterol (mmol/L) | 18 | -0.5 (-0.8, -0.2) | 24 | -0.0 (-0.4, 0.4) | 21 | 0.2 (-0.1, 0.4) | 0.027 |
| Fasting TG (mmol/L) | 18 | -0.3 (-0.5, -0.1) | 24 | -0.2 (-0.4, -0.0) | 21 | -0.2 (-0.4, -0.0) | 0.661 |
| Fasting HDL (mmol/L) | 18 | -0.1 (-0.2, 0.0) | 24 | -0.1 (-0.1, 0.0) | 21 | -0.0 (-0.1, 0.1) | 0.572 |
| Fasting LDL (mmol/L) | 18 | -0.3 (-0.6, -0.0) | 24 | 0.1 (-0.2, 0.4) | 21 | 0.3 (0.1, 0.5) | 0.012 |
| Fasting Leptin  (mg/L)^a^ | 14 | -12.7 (-18.5, -6.9) | 16 | 1.8 (-7.3, 3.6) | 16 | -1.7 (-7.2, 3.7) | 0.009 |
| Systolic BP (mmHg) | 16 | -3.3 (-10.1, 3.5) | 23 | 0.8 (-4.1, 5.7) | 20 | -2.0 (-9.3, 5.4) | 0.623 |
| Diastolic BP (mmHg) | 16 | -1.7 (-5.4, 2.1) | 23 | 1.1 (-1.1, 3.2) | 20 | 3.6 (0.5, 6.7) | 0.044 |
| HOMA-IR | 17 | -0.21(-0.46, 0.05) | 24 | -0.23(0.42,0.004) | 21 | -0.21 (-0.40, -0.02) | 0.984 |
| Omega-3 index (%) | 15 | -0.2 (-0.8, 0.4) | 23 | -0.0 (-0.5, 0.4) | 21 | 0.8 (0.1, 1.6) | 0.038 |

^a^ Leptin adjusted for % body fat as determined by DEXA
